# Cyclic alternating pattern in sleep electroencephalography as a novel predictor of dementia: A prospective study

**DOI:** 10.1002/alz.71331

**Published:** 2026-04-01

**Authors:** Ying Zheng, Xinyue Wu, Haizhen Chen, Fan Li, Kristine Yaffe, Katie Stone, Yue Leng, Xiao Tan

**Affiliations:** ^1^ Department of Psychiatry Sir Run Run Shaw Hospital Zhejiang University School of Medicine Hangzhou China; ^2^ School of Public Health Zhejiang University School of Medicine Hangzhou China; ^3^ School of Biomedical Engineering Faculty of Medicine Dalian University of Technology Dalian China; ^4^ Department of Psychiatry and Behavioral Sciences University of California San Francisco San Francisco California USA; ^5^ Departments of Neurology and Epidemiology University of California San Francisco San Francisco California USA; ^6^ Department of Research Institute California Pacific Medical Center San Francisco California USA; ^7^ Department of Clinical Neuroscience Karolinska Institutet Stockholm Sweden

**Keywords:** cohort study, cyclic alternating pattern, dementia, non‐rapid eye movement, sleep

## Abstract

**INTRODUCTION:**

Cyclic alternating pattern (CAP) is a sleep physiological rhythm observed during non–rapid eye movement (NREM) stage. However, the potential role of CAP for risk of dementia remains unknown.

**METHODS:**

We studied 2,557 participants enrolled in the prospective Osteoporotic Fractures in Men (MrOS) Sleep Study. CAP features were calculated from electroencephalogram (EEG). The primary outcomes are probable dementia determined by physician diagnosis, Alzheimer's medication use, or a significant cognitive decline.

**RESULTS:**

Participants in the lowest tertile of A2 index exhibited an increased risk of dementia compared to those in the highest tertile (hazard ratio [HR] = 2.00, 95% confidence interval [CI]: 1.38–2.90). Further, a lower combined A2+A3 index and shorter CAP sequence duration were associated with a higher risk of dementia. The Shapley Additive Explanations method indicates that CAP features were more predictive of dementia risk than conventional sleep parameters.

**DISCUSSION:**

These findings suggest that specific CAP features may serve as useful predictors to identify those older men at risk of dementia.

## Highlights

- Older men with lower A2 index, lower combined A2+A3 index, and shorter cyclic alternating pattern (CAP) sequence duration had greater risk of dementia.
- CAP features were more predictive of dementia risk than conventional sleep parameters in machine learning models.
- Specific CAP features might be useful as early predictors of dementia in the elderly.

## 1 BACKGROUND

Sleep plays a critical role in maintaining human cognitive function, and a growing body of evidence indicates that sleep disturbances are risk factors for cognitive impairment,^1^, ^2^, ^3^ including Alzheimer's disease (AD).^4^, ^5^, ^6^ Different stages of sleep may differentially affect features of AD pathophysiology.^7^ For instance, slow‐wave sleep (SWS) during the non‐rapid eye movement (NREM) sleep stage are linked with amyloid‐beta (A*β*) levels, a key pathological hallmark of AD.^8^, ^9^, ^10^, ^11^ Emerging evidence suggests that reduced spindles during NREM stage 2 (N2) sleep are negatively correlated with tau in the cerebrospinal fluid.^12^ Nonetheless, the impact of sleep microstructure on the neurodegenerative processes have been understudied. As dementia has emerged as one of the most significant global public health challenges, there is an urgent need to identify early, noninvasive biomarkers that can predict dementia before the onset of clinical symptoms.

Cyclic alternating pattern (CAP) is a sleep physiological rhythm mainly observed during NREM sleep and characterized by alternating phases of transient events (phase A) and background activity (phase B).^13^ Phase A activities can be further classified into three subtypes. Subtype A1, which is predominated by synchronized electroencephalogram (EEG) activity, has been closely associated with the buildup and consolidation of SWS. In contrast, subtypes A2 and A3 are related to the modulation of REM sleep onset.^14^ In response to environmental disturbances, the CAP system can provide a natural “slow wave injection” via a short‐term homeostatic mechanism, in which the proportion of A phases or length of CAP time is flexibly modulated to preserve sleep continuity.^15^, ^16^, ^17^

Recently, various EEG‐derived features—such as sleep spindles, slow oscillations, and spectral power—have been linked to cognitive performance and neurodegeneration in older adults.^18^, ^19^, ^20^ Despite these advances, CAP, as a key sleep microstructural feature, remains largely unknown.^4^, ^21^, ^22^ In addition, only a few cross‐sectional studies have examined the potential role of CAP in neurodegenerative processes. CAP rate and its slow components (A1 index) were reduced in patients with mild cognitive impairment (MCI) and AD, compared to cognitively normal controls.^23^, ^24^ Further, after a 2‐year follow‐up of amnestic MCI individuals, those who converted to AD exhibited a greater decrease in CAP rate, A1 index, and A3 index compared with non‐converters.^24^ To date, no prospective cohort study has evaluated whether CAP features, particularly subtypes of phase A, are associated with the risk of incident dementia. As a key component of sleep microstructure, CAP may represent novel predictors for detecting early‐phase cognitive impairment.

The purpose of this study was to investigate whether CAP characteristics measured through overnight polysomnography at baseline were associated with incident dementia over long‐term follow‐up in a well‐characterized community‐based cohort. We hypothesized that reduced A phase activity and altered CAP rate may be associated with increased dementia risk. To further compare the relative importance of CAP characteristics, we additionally included conventional sleep parameters to develop a predictive model using a machine learning approach. Our findings may provide new insights into the utility of sleep microstructure as a novel predictor for dementia.

## 2 METHODS

### 2.1 Study participants

The Osteoporotic Fractures in Men (MrOS) Study (https://mrosonline.ucsf.edu (July 15 2025)) is a prospective cohort study of 5994 community‐dwelling men 65 years or older from six clinical centers throughout the United States.^25^, ^26^Between December 2003 and March 2005, the MrOS Sleep study recruited 3,135 participants from the initial cohort to undergo an overnight polysomnography (baseline of this study). Among them, 2,967 were free of dementia at baseline and 2,904 had available EEG data. 2,586 were included in the final analysis after excluding those with missing key information. Detailed information on participant selection is shown in Supplemental Figure **1**. All participants provided written informed consent, and the protocol was approved by Institutional Review Boards at each individual site.

**RESEARCH IN CONTEXT**

1. **Systematic review**: The authors reviewed the literature with PubMed using search terms related to sleep microstructure, cyclic alternating pattern (CAP), sleep electroencephalography and dementia. While only limited cross‐sectional evidence has linked CAP features to cognitive impairment, no study has focused on the longitudinal association between CAP and dementia.
2. **Interpretation**: Our findings indicate that lower A2 index, lower combined A2+A3 index, and shorter CAP sequence duration was associated with a higher risk of dementia over 9 years in older men. These findings suggest that specific CAP features may serve as useful predictors to identify those at elevated risk of dementia.
3. **Future directions**: Future studies with repeatedly measured CAP are required to evaluate the longitudinal evolution of CAP on brain aging. Randomized controlled trials are warranted to determine whether treatments targeting CAP can modify the risk of dementia.

### 2.2 Assessment of CAP

One night of at‐home unattended polysomnography (PSG) was performed at the Sleep Visit 1. Staff returned the next morning to collect the equipment and download the data, which was transferred to the Central Sleep Reading Center for centralized scoring by a trained technician according to standard criteria.^27^, ^28^ Details can be found elsewhere.^29^, ^30^ We processed raw EEG timeseries data with MATLAB R2021b (MathWorks, Inc., Natick, MA). EEG was recorded from the central recording locations C3 and C4 using gold cup electrodes, originally recorded with an Fpz reference, re‐referenced to a contralateral mastoid reference. Thus, two EEG channels were used, which were C3‐A2 and C4‐A1. We applied a previously developed, highly precise automated system for CAP analysis, which is described in the work of Hartmann and Baumert in detail.^31^, ^32^ This algorithm is based on a deep learning recurrent neural network (RNN) framework that was trained specifically to recognize A‐phases in EEG recordings, achieving improved accuracy and sensitivity compared with previously proposed systems.^33^ Briefly, the system is divided into four major parts: preprocessing, feature extraction, classification, and post‐processing. The data were resampled at a frequency of 128 Hz to remove the cardiac field and eye movement artifacts. Before extracting signal features, the EEG was bandpass filtered with a finite impulse response (FIR) filter (0.5 Hz–30 Hz) and subsequently divided into five frequency bands. The event‐based inter‐rater reliability between human scorers ranges from 0.42 and 0.75, whereas the second‐by‐second A‐phase inter‐rater reliability between visual scoring and this system was between 0.53 and 0.56, as quantified by the Cohen's kappa coefficient, indicating reliable agreement between the system and manual scoring.^34^ The following CAP parameters were calculated: CAP rate (total CAP time / total NREM time × 100), CAP index (number of CAP sequences / hours of NREM sleep), A1/A2/A3 index (number of specific phase A subtype [A1/A2/A3] / hours of NREM sleep), the number of CAP sequences, mean CAP sequence duration. In addition to the individual A subtype index, we constructed a composite A2+A3 index to provide a more integrative assessment of arousal‐related CAP activity, as both A2 and A3 phases constitute a central nervous system arousal and are closely involved in REM‐on regulation.^13^, ^35^

### 2.3 Ascertainment of dementia

The outcome in our study was probable dementia at any follow‐up visit after baseline (Sleep Visit 1), defined by a report of physician‐diagnosed dementia, use of dementia medication, or a change in Modified Mini‐Mental State Examination (3MS) score ≥ 1.5 standard deviations worse than the mean change from baseline to any follow‐up visit. The follow‐up time was calculated from Sleep Visit 1 where PSG was measured to the earliest occurrence of dementia, death, or the end of follow‐up (Visit 4).

### 2.4 Covariates

All participants completed questionnaires at baseline, which included items about age, ethnicity (categorized as White or other), education (categorized as high school or less and college or higher, smoking status (categorized as current, former and never), alcohol consumption (categorized as yes or no). Body mass index (BMI) was calculated as weight in kilograms divided by the square of height in meters. Level of physical activity was assessed using the Physical Activity Scale for the Elderly (PASE).^36^ Total sleep time (TST), wake after sleep onset (WASO), NREM stage percentage and Apnea–Hypopnea Index (AHI) (3% rule for hypopnea) were derived from the traditional manual sleep scoring from PSG. Hypertension and diabetes status was determined by self‐reported medical history. Participants were asked to bring in all medications used within the preceding 30 days. All prescription and nonprescription medications were entered into an electronic database and each medication was matched to its ingredient(s) based on the Iowa Drug Information Service Drug Vocabulary (College of Pharmacy, University of Iowa, Iowa City, IA).^37^ The use of antidepressants, benzodiazepines, and other sleep medications (non‐benzodiazepines, nonbarbiturate sedative hypnotics) were obtained.

### 2.5 Statistical analysis

All categorical variables were presented as *n* (%), and continuous variables were expressed as mean ± SD or median (interquartile range), depending on the distribution of variables. CAP rate, CAP index, A1 to A3 index, the number of CAP sequences and mean CAP sequence duration were divided into tertiles to evaluate the association between CAP and dementia. The percentage of missing values are present in Supplementary Table **1**. Cox proportional hazard models were used to examine the associations between CAP and risk of incident dementia, and were tested using the Schoenfeld residual method. We fit two models: a crude model (adjusting for age) and a fully adjusted model (additionally adjusting for ethnicity, BMI, education, TST, WASO, history of diabetes and hypertension, smoking status, alcohol consumption, PASE score, the use of antidepressants, benzodiazepines, and other sleep medications).

Stratified analyses were performed by age (< 75 years or ≥75 years), BMI (< 30.0 or ≥30.0), and the existence of T2D and hypertension (yes or no), and interaction terms were tested using likelihood ratio test. Sensitivity analyses were conducted to assess the robustness of the main findings of our study. We conducted a Fine‐Grey proportional hazards regression to investigate the association between CAP features and dementia after controlling for all confounders, considering the competing risk of death. We also adjusted for additional covariates: NREM stage percentage and AHI. Moreover, Spearman's correlation was used for correlation analysis between CAP features and conventional sleep parameters.

Additionally, the dataset was divided into training and testing sets in a 7:3 proportion and we employed a machine learning algorithms—eXtreme Gradient Boosting (XGBoost)—to develop a prediction model. In the machine learning model, we included the same covariates of the Cox regression model to ensure consistency for dementia risk prediction. To maximize the accuracy of XGBoost, we ran a hyperparameter grid search using 10‐fold cross‐validation with receiver operating characteristic (ROC) curves serving as the primary criterion for selecting optimal parameter values to determine the final parameter combinations. Subsequently, the trained models were validated on the respective testing sets, and performance was assessed using a suite of evaluation metrics: ROC curve area under the curve (AUC) ranging from 0 to 1, where higher values indicate superior model performance. To further elucidate the determinants of dementia risk, we applied SHapley Additive exPlanation (SHAP) values to understand and visualize the importance of predictive features in the optimal model. STATA 16.0 and Anaconda python 3 version 3.8 were used for statistical analysis and computing. A two‐sided *p*‐value of < 0.05 was regarded as statistically significant.

## 3 RESULTS

### 3.1 Baseline characteristics

A total of 2,557 participants were included in the study. Baseline characteristics stratified by CAP rate tertile are shown in Table 1. Overall, baseline participants had a mean age of 76.2 years (standard deviation, 5.4 years) and 90.7% were White individuals. Compared to those in the highest tertile of CAP rate, participants in the lowest tertile were older, had higher BMI, higher frequency of alcohol consumption and higher use of antidepressants, benzodiazepines, and sleep medications, and exhibited a higher prevalence of hypertension and diabetes. They were less likely to be educated, of White ethnicity and be a current smoker.

**TABLE 1:** Baseline characteristics of the participants

		CAP rate			
Characteristics	Total (*N* = 2,557)	Tertile 1 (*N* = 853)	Tertile 2 (*N* = 852)	Tertile 3 (*N* = 852)	*p*‐Value
Age (years), median (IQR)	75 (72–80)	76 (72–80)	76 (72–80)	75 (71–79)	0.002
Ethnicity, *n* (%)					0.462
White	2319 (90.7)	765 (89.7)	777 (91.2)	777 (91.2)	
Education, *n* (%)					0.460
College or higher	2,023 (79.1)	663 (77.7)	682 (80.0)	678 (79.6)	
BMI (kg/m^2^), median (IQR)	27 (25–29)	27 (25–30)	27 (25–29)	26 (24–29)	< 0.001
PASE score, median (IQR)	151 (109.1–195)	146.2 (108.1–192.1)	157.4 (113.6–194.8)	149.5 (106.8–197.4)	0.288
Smoking, *n* (%)					0.389
Never	1,031 (40.3)	328 (38.4)	344 (40.4)	359 (42.1)	
Former	1,476 (57.7)	504 (59.1)	495 (58.1)	477 (56.0)	
Current	50 (1.9)	21 (2.5)	13 (1.5)	16 (1.9)	
Alcohol, *n* (%)					0.297
Alcohol consumer	1696 (66.3)	549 (64.4)	569 (66.8)	578 (67.8)	
Hypertension, *n* (%)	1,262 (49.3)	450 (52.7)	432 (50.7)	380 (44.6)	0.002
Diabetes, *n* (%)	331 (12.9)	144 (16.9)	101 (11.8)	86 (10.1)	< 0.001
TST (mins/day), median (IQR)	362 (320–401)	368 (325–410)	363 (323–403)	357 (313.5–392.5)	< 0.001
WASO (mins/day), median (IQR)	101 (66–148)	104 (67–152)	99 (66–148)	99 (65–144)	0.237
AHI (/hour), median (IQR)	17 (9–28)	15 (8–28)	16 (9–27)	18 (9–31)	0.008
NREM percentage (%), mean ± SD	80.6 ± 6.5	80.9 ± 6.7	80.5 ± 6.4	80.6 ± 6.3	0.287
N1 percentage (%), median (IQR)	6 (4–8)	6 (4–8)	6 (4–9)	6 (4–8)	0.533
N2 percentage (%), median (IQR)	63 (56–69)	64 (58–71)	63 (56–69)	62 (55–68)	< 0.001
SWS percentage (%), median (IQR)	10 (4–17)	8 (3–15)	10 (4–17)	12 (5–18)	< 0.001
REM percentage (%), mean ± SD	19.3 ± 6.5	19.1 ± 6.7	19.5 ± 6.4	19.4 ± 6.3	0.483
Use of antidepressants, *n* (%)	187 (7.3)	72 (8.4)	64 (7.5	51 (6.0)	0.145
Use of benzodiazepines, *n* (%)	107 (4.2)	64 (12.5)	20 (2.3)	23 (2.7)	< 0.001
Use of other sleep medications, *n* (%)	53 (2.1)	26 (3.4)	13 (1.5)	14 (1.6)	0.049

Abbreviations: AHI, Apnea–Hypopnea Index; BMI, body mass index; CAP, cyclic alternating pattern; IQR, interquartile range; NREM, non‐rapid eye movement; PASE, Physical Activity Scale for the Elderly; REM, rapid eye movement; SWS, slow‐wave sleep; TST, total sleep time; WASO, wake after sleep onset.

### 3.2 Associations between CAP features and dementia

During a median follow‐up of 9.0 years, 173 participants developed incident dementia. Compared to participants in the highest tertile of A2 index, those in the lowest tertile of A2 index had a 104% higher risk of developing dementia after adjustment for age, ethnicity, BMI, education, TST, WASO, history of diabetes and hypertension, smoking status, alcohol consumption, PASE score, and the use of antidepressants, benzodiazepines, and other sleep medications (hazard ratio [HR] 2.04, 95% confidence interval [CI]: 1.41–2.96, Table 2). Similar patterns were observed in the association between A2+A3 index and incident dementia (HR 1.91, 95% CI: 1.29–2.82, lowest vs. highest tertile). Lower mean CAP sequence duration was associated with higher risk of incident dementia. However, no association of CAP rate, CAP index, the number of CAP sequences, A1 index and A3 index with incident dementia was detected after multivariate adjustment.

**TABLE 2,:** Association of CAP with the risk of dementia.

Parameter	No. of cases	No. of participants	Crude model ^a^	Fully adjusted model ^b^
CAP rate (%) cap time/NREM time				
T1	67	853	1.32 (0.92, 1.90)	1.36 (0.94, 1.98)
T2	55	852	1.07 (0.73, 1.57)	1.08 (0.73, 1.58)
T3	51	852	1	1
*p*‐value for trend			0.112	0.083
CAP index (cap cycles/h)				
T1	64	853	1.28 (0.88, 1.84)	1.34 (0.92, 1.95)
T2	58	852	1.15 (0.79, 1.68)	1.14 (0.78, 1.67)
T3	51	852	1	1
*p*‐value for trend			0.310	0.166
No. of CAP sequences				
T1	65	915	0.99 (0.70, 1.41)	1.03 (0.71, 1.49)
T2	47	792	0.82 (0.56, 1.21)	0.83 (0.57, 1.23)
T3	61	850	1	1
*p*‐value for trend			0.458	0.334
CAP sequence duration, min				
T1	65	854	1.22 (0.85, 1.76)	1.22 (0.84, 1.76)
T2	56	851	1.08 (0.74, 1.58)	1.16 (0.79, 1.70)
T3	52	852	1	1
*p*‐value for trend			0.032	0.025
A1 index				
T1	59	853	0.97 (0.68, 1.40)	1.01 (0.70, 1.45)
T2	54	852	0.87 (0.60, 1.26)	0.89 (0.61, 1.28)
T3	60	852	1	1
*p*‐value for trend			0.476	0.630
A2 index				
T1	82	853	1.02 (1.32, 2.78)	2.00 (1.38, 2.90)
T2	48	852	1.11 (0.74, 1.07)	1.14 (0.75, 1.72)
T3	43	852	1	1
*p*‐value for trend			< 0.001	< 0.001
A3 index				
T1	60	853	1.24 (0.85, 1.81)	1.26 (0.86, 1.84)
T2	63	852	1.28 (0.88, 1.85)	1.27 (0.87, 1.84)
T3	50	852	1	1
*p*‐value for trend			0.135	0.104
A2 + A3 index				
T1	70	853	1.81 (1.23, 2.67)	1.87 (1.26, 2.76)
T2	63	852	1.61 (1.08, 2.39)	1.60 (1.08, 2.40)
T3	40	852	1	1
*p*‐value for trend			0.009	0.005

^a^ Crude model was adjusted for age.

^b^ Fully adjusted model was further adjusted for ethnicity, BMI, education, TST, WASO, history of diabetes and hypertension, smoking status, alcohol consumption, PASE score, the use of antidepressants, benzodiazepines, and other sleep medications.

Abbreviations: BMI, body mass index; CAP, cyclic alternating pattern; NREM, non‐rapid eye movement; PASE, Physical Activity Scale for the Elderly; TST, total sleep time; WASO, wake after sleep onset

### 3.3 Subgroup and sensitivity analyses

Consistent results were observed when analyses were stratified by age, BMI and the existence of T2D (Table S2–S4). When stratified by the existence of hypertension, lower CAP rate, CAP index, A2 index, A3 index, and A2+A3 index were significantly associated with dementia among participants with hypertension, with evidence of interaction between hypertension and CAP rate, number of CAP sequence, mean CAP sequence duration, A2 index, and A3 index (Table S5). The associations between CAP features and risk of dementia did not substantially change when death was treated as a competing risk for dementia and additionally adjustment for NREM percentage and AHI (Table S6–S7). Spearman correlation analyses revealed generally low correlations between CAP features and conventional sleep measures (TST, WASO, AHI, NREM%) (Table S8).

### 3.4 Predictive performance

XGboost algorithm and SHAP values were used to understand the importance of individual CAP features to the prediction model (Figure 1 and Figure S2). While the predictive performance of the model was modest (Table S9), machine learning models suggested better predictive performance of CAP features than conventional sleep parameters, such as TST, WASO, NREM percentage, and AHI.

FIGURE 1SHAP values from an XGBoost model trained to predict the risk of dementia. (A) CAP rate; (B) CAP index; (C) No. of CAP sequences; (D) CAP sequence duration; (E) A1 index; (F) A2 index; (G) A3 index; (H) A2+A3 index. Features are ordered based on their cumulative effect on model output. BMI, body mass index; PASE, physical activity scale for the elderly; SHAP, SHapley Additive exPlanation; TST, total sleep duration; WASO, wake after sleep onset.

**Figure:**
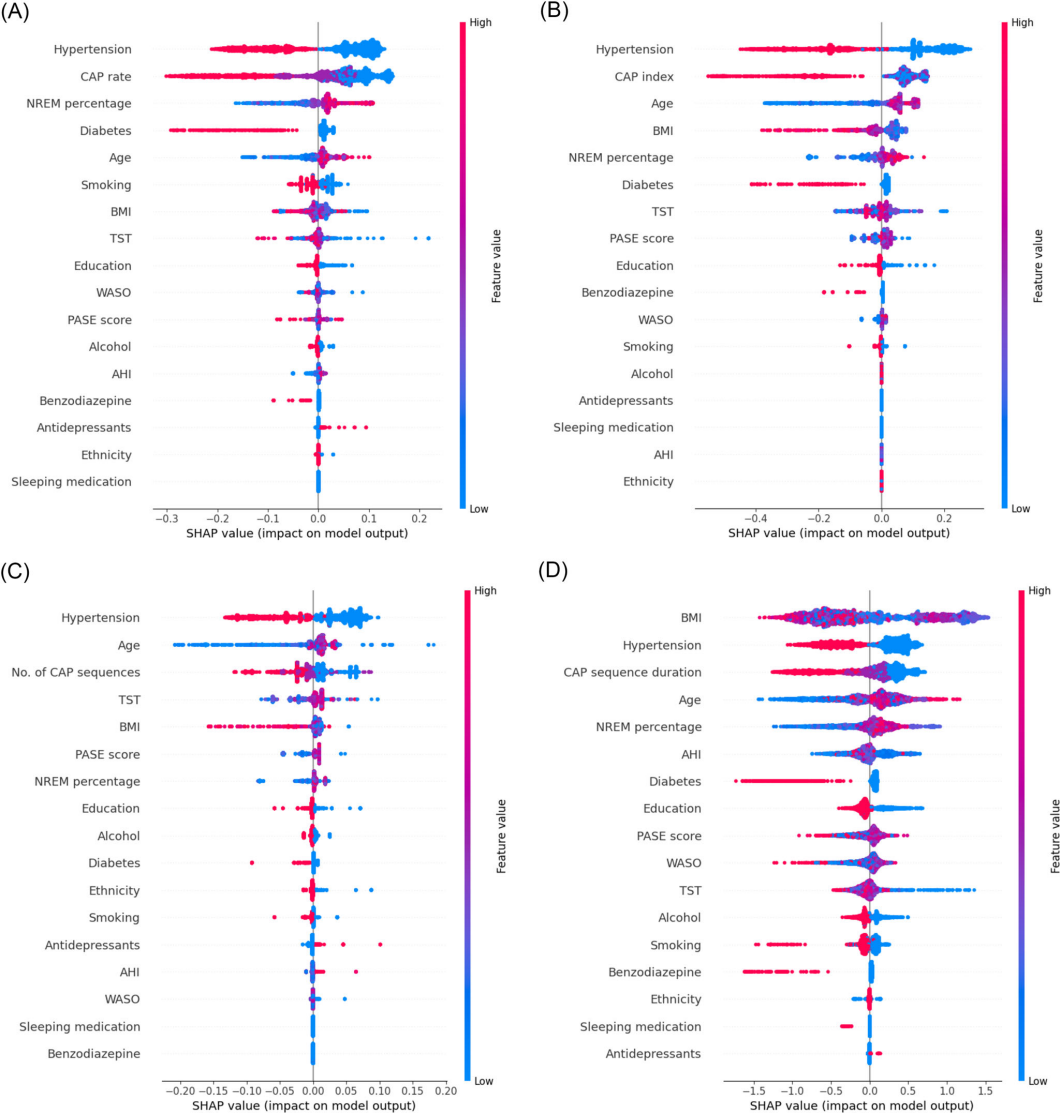

**Figure:**
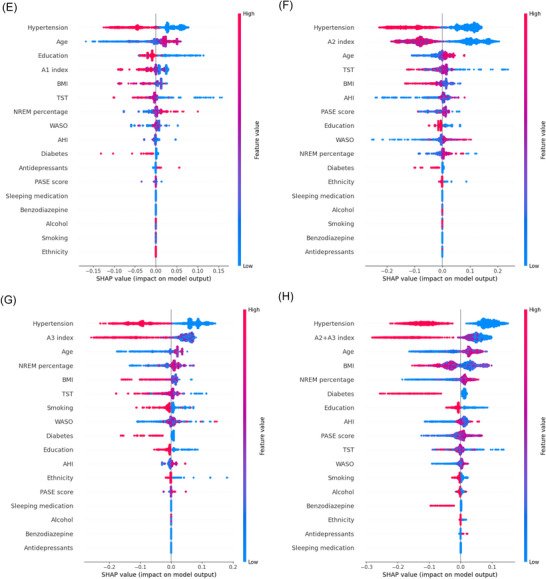

## 4 DISCUSSION

To our knowledge, this study is the first to investigate the association between CAP and incident dementia. In this prospective study of 2,557 older men, we found that A2 index, either alone or in combination with A3 index, was associated with incident dementia. Our findings also provided novel evidence on these less frequently investigated sleep microstructural parameters, highlighting the potential value of integrating multiple sleep metrics into dementia risk prediction using innovative machine learning approaches. Notably, specific CAP features appeared more sensitive and predictive than traditional sleep measures (e.g., TST, WASO, NREM percentage, and AHI).

Previous studies have been limited to a cross‐sectional design comparing CAP features in individuals with MCI or AD to cognitively intact controls.^23^, ^24^ In this study, participants with lower A2 index exhibited a higher risk of dementia, which was supported by the established research showing that compared with healthy controls, subjects with MCI showed a decreased A2 index.^24^ Similar pattern was found for A2+A3 pattern, suggesting that specific CAP features may serve as early predictor of dementia progression. Further, we found that lower mean CAP sequence duration was associated with higher risk of incident dementia. A recent study developing a sleep EEG‐based brain age found that transient, high‐amplitude events were inversely related to incident dementia, potentially reflecting underlying CAP dynamics, which suggested that the temporal organization of CAP and non‐CAP activity could represent a fundamental, yet underappreciated, aspect of sleep microstructure relevant to dementia pathophysiology.^38^ Collectively, these findings suggest that sleep microstructure may offer important insights into the pathophysiological process of neurodegeneration, and the role of CAP rate and sequence duration in the risk of dementia remains to be further investigated.^39^ While no overall association between CAP rate and incident dementia was observed, stratified analyses revealed that among individuals with hypertension, those in the lowest tertile of CAP rate had an elevated risk of dementia compared with those in the highest tertile. The observed interaction between CAP and hypertension may reflect a complex interplay between sympathetic excitation and baroreflex regulation during sleep. Sustained sympathetic activation occurring in CAP was accompanied by an increase in baroreflex sensitivity (BRS) serving to buffer blood pressure surge, which is frequently impaired among individuals with hypertension, resulting in exaggerated nocturnal blood pressure variability.^40^, ^41^ Such abnormal blood pressure dynamics may promote cerebral hypoperfusion and microvascular dysfunction, ultimately accelerating neurodegenerative processes and increasing dementia risk.^42^, ^43^, ^44^, ^45^

These results extend a growing body of research linking sleep disruption to AD. Prior studies have shown that accelerated deterioration in sleep EEG microstructure, such as spectral power and spindle‐slow oscillation coupling, is associated with cognitive impairment and dementia.^18^, ^38^, ^46^ Mechanistically, the association between A2 index and incident dementia may be explained by disruption of SWS, which is related to oscillation of CAP.^16^, ^47^, ^48^, ^49^ Notably, A2 typically begins with slow waves, and sufficient high‐quality SWS plays a pivotal role in metabolic clearance and memory consolidation.^15^, ^50^, ^51^ Reduced SWS sleep has been consistently associated with greater A*β* burden, one of the most common pathogeneses of AD.^9^, ^52^Disrupted SWS may facilitate a bidirectional cycle in which increased A*β* aggregation further impairs SWS.^7^ Meanwhile, slow‐wave activity (SWA) has been proposed to restore optimal learning capacity through downscaling and renormalization of synaptic strength, particularly under conditions of elevated A*β* burden, thus representing a critical mechanistic bridge between sleep alteration and neurodegeneration.^53^, ^54^, ^55^ Furthermore, age‐related reductions in SWS may lead to sustained impairment of glymphatic clearance, promoting amyloid plaque deposition and cortical tau propagation, thereby accelerating neurodegeneration and dementia development.^56^, ^57^ In addition, the link between lower A2 index and increased risk of dementia may result from REM sleep disturbances, as A2 modulated the onset of REM sleep. Evidence suggests that reduced REM duration and prolonged REM latency were associated with accumulation and neurodegeneration in aging populations.^58^, ^59^, ^60^ These alterations may arise from early impairment of basal forebrain cholinergic networks and neurotransmitter imbalance, which are central to REM sleep physiology.^61^, ^62^, ^63^ Dysregulation of the orexinergic system may further destabilize the REM‐wake balance by promoting hyperarousal and suppressing REM onset.^64^, ^65^ Beyond neurochemical control, REM sleep integrity is also shaped by sleep–wake homeostasis and circadian timing, which are essential for A*β* and tau clearance.^29^, ^66^, ^67^ Although the full connection between sleep micro‐architecture and dementia remains incompletely understood, CAP loss appears as a predictor of a stage in disease progression.^29^, ^48^, ^68^

Our findings have important clinical and public health implications. By leveraging advanced machine learning techniques and SHAP‐based interpretation, our results highlight the role of CAP features in dementia risk prediction. PSG‐derived sleep markers may serve as a complementary and noninvasive tool for identifying individuals at elevated risk of dementia and MCI, thereby enabling clinicians to implement targeted sleep interventions. Collectively, our results advance the precision public health agenda by emphasizing the importance of integrating sleep microstructure into predictive modeling and prevention strategies for dementia.

A significant strength of our study is the use of objective sleep measures from PSG, which offers more accurate and comprehensive measurement of sleep. Moreover, a machine learning approach was used to compare and rank the important features that may contribute to the predictive model, and further confirmed the role of CAP features in the prediction of dementia. Nevertheless, the study was subject to several limitations. First, sleep was only measured over a single night, which may not reflect the long‐term CAP features. Repeated sleep assessments would provide stronger predictive power. Second, the diagnosis of dementia was based on self‐reported data, which may cause misclassification of outcome. Third, the timing of dementia incidence relied on study visit dates and may not accurately reflect the actual onset of dementia. Fourth, the sample mainly involved older White men, limiting the generalizability of the findings. Future research should include more diverse populations. Fifth, though we adjusted for many covariates, residual confounding and unobserved factors (e.g., subclinical disease) cannot be entirely ruled out. To address potential reverse causation, we excluded participants with significant cognitive impairment at baseline based on a 3MS score of less than 80 or use of dementia medication. Lastly, although the automated CAP detection algorithm used in the present study showed excellent performance compared with manual scoring, its accuracy depends on the training data, potentially introducing scorer‐specific biases.^31^ Given that visual scoring is time‐consuming for large sample size, further work is warrant to validate and refine automated methods for CAP scoring to support broader research and clinical applications.

In summary, analysis of sleep microarchitecture like CAP features provides a unique window for better understanding of dementia risk. Our results emphasize the potential utility of CAP assessment to help with the early detection and prevention of dementia.

## CONFLICT OF INTEREST STATEMENT

Dr. Stone discloses funding from Eli Lilly, Axsome Therapeutics, and Sleep Research Society, outside of the current work. All other authors declare no competing interests. Author disclosures are available in the Supporting Information.

## CONSENT STATEMENT

All men provided written informed consent.

## Supporting information



Supporting Information

Supporting Information

## References

[1] Fernandez‐Mendoza J , He F , Puzino K , et al. Insomnia with objective short sleep duration is associated with cognitive impairment: a first look at cardiometabolic contributors to brain health. Sleep. 2021;44:zsaa150. 32968796 10.1093/sleep/zsaa150 PMC7819842

[2] Keil SA , Schindler AG , Wang MX , et al. Longitudinal sleep patterns and cognitive impairment in older adults. JAMA Netw Open. 2023;6:e2346006. 38048131 10.1001/jamanetworkopen.2023.46006 PMC10696486

[3] Qin S , Leong RL , Ong JL , Chee MW . Associations between objectively measured sleep parameters and cognition in healthy older adults: a meta‐analysis. Sleep Med Rev. 2023;67:101734. 36577339 10.1016/j.smrv.2022.101734

[4] Sabia S , Fayosse A , Dumurgier J , et al. Association of sleep duration in middle and old age with incidence of dementia. Nat Commun. 2021;12:2289. 33879784 10.1038/s41467-021-22354-2 PMC8058039

[5] Shi L , Chen S‐J , Ma M‐Y , et al. Sleep disturbances increase the risk of dementia: a systematic review and meta‐analysis. Sleep Med Rev. 2018;40:4‐16. 28890168 10.1016/j.smrv.2017.06.010

[6] Xiong Y , Tvedt J , Akerstedt T , Cadar D , Wang HX . Impact of sleep duration and sleep disturbances on the incidence of dementia and Alzheimer's disease: a 10‐year follow‐up study. Psychiatry Res. 2024;333:115760. 38301285 10.1016/j.psychres.2024.115760

[7] Ju Y‐ES , Lucey BP , Holtzman DM . Sleep and Alzheimer disease pathology—a bidirectional relationship. Nat Rev Neurol. 2014;10:115‐119. 24366271 10.1038/nrneurol.2013.269 PMC3979317

[8] Winer JR , Mander BA , Kumar S , et al. Sleep disturbance forecasts *β*‐amyloid accumulation across subsequent years. Curr Biol. 2020;30:4291‐4298. e3. 32888482 10.1016/j.cub.2020.08.017 PMC7642104

[9] Mander BA , Winer JR , Jagust WJ , Walker MP . Sleep: a novel mechanistic pathway, biomarker, and treatment target in the pathology of Alzheimer's disease?. Trends Neurosci. 2016;39:552‐566. 27325209 10.1016/j.tins.2016.05.002 PMC4967375

[10] Wunderlin M , Züst MA , Fehér KD , Klöppel S , Nissen C . The role of slow wave sleep in the development of dementia and its potential for preventative interventions. Psychiatry Res Neuroimaging. 2020;306:111178. 32919869 10.1016/j.pscychresns.2020.111178

[11] Varga AW , Wohlleber ME , Giménez S , et al. Reduced slow‐wave sleep is associated with high cerebrospinal fluid A*β*42 levels in cognitively normal elderly. Sleep. 2016;39:2041‐2048. 27568802 10.5665/sleep.6240 PMC5070758

[12] Kam K , Parekh A , Sharma RA , et al. Sleep oscillation‐specific associations with Alzheimer's disease CSF biomarkers: novel roles for sleep spindles and tau. Mol Neurodegener. 2019;14:10. 30791922 10.1186/s13024-019-0309-5 PMC6385427

[13] Parrino L , Ferri R , Bruni O , Terzano MG . Cyclic alternating pattern (CAP): the marker of sleep instability. Sleep Med Rev. 2012;16:27‐45. 21616693 10.1016/j.smrv.2011.02.003

[14] Terzano MG , Parrino L , Smerieri A , et al. CAP and arousals are involved in the homeostatic and ultradian sleep processes. J Sleep Res. 2005;14:359‐368. 16364136 10.1111/j.1365-2869.2005.00479.x

[15] Halász P , Bódizs R , Parrino L , Terzano M . Two features of sleep slow waves: homeostatic and reactive aspects‐from long term to instant sleep homeostasis. Sleep Med. 2014;15:1184‐1195. 25192672 10.1016/j.sleep.2014.06.006

[16] Parrino L , Vaudano AE . The resilient brain and the guardians of sleep: new perspectives on old assumptions. Sleep Med Rev. 2018;39:98‐107. 29054694 10.1016/j.smrv.2017.08.003

[17] Carra MC , Macaluso GM , Rompré PH , et al. Clonidine has a paradoxical effect on cyclic arousal and sleep bruxism during NREM sleep. Sleep. 2010;33:1711‐1716. 21120152 10.1093/sleep/33.12.1711 PMC2982742

[18] Djonlagic I , Mariani S , Fitzpatrick AL , et al. Macro and micro sleep architecture and cognitive performance in older adults. Nat Hum Behav. 2021;5:123‐145. 33199858 10.1038/s41562-020-00964-y PMC9881675

[19] Ujma PP , Bódizs R , Dresler M , et al. Multivariate prediction of cognitive performance from the sleep electroencephalogram. Neuroimage. 2023;279:120319. 37574121 10.1016/j.neuroimage.2023.120319 PMC10661862

[20] Páez A , Gillman SO , Dogaheh SB , et al. Sleep spindles and slow oscillations predict cognition and biomarkers of neurodegeneration in mild to moderate Alzheimer's disease. Alzheimers Dement. 2025;21:e14424. 39878233 10.1002/alz.14424 PMC11848347

[21] Wang S , Zheng X , Huang J , Liu J , Li C , Shang H . Sleep characteristics and risk of Alzheimer's disease: a systematic review and meta‐analysis of longitudinal studies. J Neurol. 2024;271:3782‐3793. 38656621 10.1007/s00415-024-12380-7

[22] Tan X , Åkerstedt T , Lagerros YT , et al. Interactive association between insomnia symptoms and sleep duration for the risk of dementia—a prospective study in the swedish national march cohort. Age Ageing. 2023;52:afad163. 37676841 10.1093/ageing/afad163 PMC10484328

[23] Maestri M , Carnicelli L , Tognoni G , et al. Non‐rapid eye movement sleep instability in mild cognitive impairment: a pilot study. Sleep Med. 2015;16:1139‐1145. 26298791 10.1016/j.sleep.2015.04.027

[24] Carnicelli L , Maestri M , Di Coscio E , et al. A longitudinal study of polysomnographic variables in patients with mild cognitive impairment converting to Alzheimer's disease. J Sleep Res. 2019;28:e12821. 30724408 10.1111/jsr.12821

[25] Blank JB , Cawthon PM , Carrion‐Petersen ML , et al. Overview of recruitment for the osteoporotic fractures in men study (MrOS). Contemp Clin Trials. 2005;26:557‐568. 16085466 10.1016/j.cct.2005.05.005

[26] Orwoll E , Blank JB , Barrett‐Connor E , et al. Design and baseline characteristics of the osteoporotic fractures in men (MrOS) study‐a large observational study of the determinants of fracture in older men. Contemp Clin Trials. 2005;26:569‐585. 16084776 10.1016/j.cct.2005.05.006

[27] Scoring E . EEG arousals: scoring rules and examples: a preliminary report from the sleep disorders atlas task force of the American sleep disorders association. Sleep. 1992;15:174‐184. 11032543

[28] Redline S , Sanders MH , Lind BK , et al. Methods for obtaining and analyzing unattended polysomnography data for a multicenter study. Sleep. 1998;21:759‐767. 11300121

[29] Zhang Y , Ren R , Yang L , et al. Sleep in Alzheimer's disease: a systematic review and meta‐analysis of polysomnographic findings. Transl Psychiatry. 2022;12:136. 35365609 10.1038/s41398-022-01897-y PMC8976015

[30] Shahrbabaki SS , Linz D , Hartmann S , Redline S , Baumert M . Sleep arousal burden is associated with long‐term all‐cause and cardiovascular mortality in 8001 community‐dwelling older men and women. Eur Heart J. 2021;42:2088‐2099. 33876221 10.1093/eurheartj/ehab151 PMC8197565

[31] Hartmann S , Baumert M . Automatic a‐phase detection of cyclic alternating patterns in sleep using dynamic temporal information. IEEE Trans Neural Syst Rehabil Eng. 2019;27:1695‐1703. 31425039 10.1109/TNSRE.2019.2934828

[32] Hartmann S , Bruni O , Ferri R , Redline S , Baumert M . Characterization of cyclic alternating pattern during sleep in older men and women using large population studies. Sleep. 2020;43:zsaa016. 32022886 10.1093/sleep/zsaa016 PMC7355398

[33] Machado F , Sales F , Santos C , Dourado A , Teixeira C . A knowledge discovery methodology from EEG data for cyclic alternating pattern detection. Biomed Eng Online. 2018;17:185. 30563526 10.1186/s12938-018-0616-z PMC6299667

[34] Ferri R , Bruni O , Miano S , Smerieri A , Spruyt K , Terzano MG . Inter‐rater reliability of sleep cyclic alternating pattern (CAP) scoring and validation of a new computer‐assisted CAP scoring method. Clin Neurophysiol. 2005;116:696‐707. 15721084 10.1016/j.clinph.2004.09.021

[35] Terzano MG , Parrino L , Sherieri A , et al. Atlas, rules, and recording techniques for the scoring of cyclic alternating pattern (CAP) in human sleep. Sleep Med. 2001;2:537‐554. 14592270 10.1016/s1389-9457(01)00149-6

[36] Washburn RA , Smith KW , Jette AM , Janney CA . The physical activity scale for the elderly (PASE): development and evaluation. J Clin Epidemiol. 1993;46:153‐162. 8437031 10.1016/0895-4356(93)90053-4

[37] Pahor M , Chrischilles E , Guralnik J , Brown S , Wallace R , Carbonin P . Drug data coding and analysis in epidemiologic studies. Eur J Epidemiol. 1994;10:405‐411. 7843344 10.1007/BF01719664

[38] Sun H , Milton S , Fang Y , et al. Machine learning–based sleep electroencephalographic brain age index and dementia risk: an individual participant data meta‐analysis. JAMA Netw Open. 2026;9:e261521. 41854616 10.1001/jamanetworkopen.2026.1521 PMC13003368

[39] Chang H , Tang W , Wulf AM , et al. Sleep microstructure organizes memory replay. Nature. 2025;637:11. 39743590 10.1038/s41586-024-08340-w PMC12107872

[40] Iellamo F , Placidi F , Marciani MG , et al. Baroreflex buffering of sympathetic activation during sleep: evidence from autonomic assessment of sleep macroarchitecture and microarchitecture. Hypertension. 2004;43:814‐819. 14981054 10.1161/01.HYP.0000121364.74439.6a

[41] Legramante JM , Marciani MG , Placidi F , et al. Sleep‐related changes in baroreflex sensitivity and cardiovascular autonomic modulation. J Hypertens. 2003;21:1555‐1561. 12872051 10.1097/00004872-200308000-00021

[42] Ma Y , Zhang Y , Hamaya R , et al. Baroreflex sensitivity and long‐term dementia risk in older adults. Hypertension. 2025;82:347‐356. 39670317 10.1161/HYPERTENSIONAHA.124.24001 PMC11735285

[43] Oishi E , Ohara T , Sakata S , et al. Day‐to‐day blood pressure variability and risk of dementia in a general Japanese elderly population: the Hisayama study. Circulation. 2017;136:516‐525. 28784822 10.1161/CIRCULATIONAHA.116.025667 PMC5548511

[44] Ma Y , Wolters FJ , Chibnik LB , et al. Variation in blood pressure and long‐term risk of dementia: a population‐based cohort study. PLoS Med. 2019;16:e1002933. 31714941 10.1371/journal.pmed.1002933 PMC6850672

[45] Alpérovitch A , Blachier M , Soumaré A , et al. Blood pressure variability and risk of dementia in an elderly cohort, the three‐city study. Alzheimers Dement. 2014;10:S330‐S7. 23954028 10.1016/j.jalz.2013.05.1777

[46] Lam AKF , Carrick J , Kao CH , et al. Electroencephalographic slowing during REM sleep in older adults with subjective cognitive impairment and mild cognitive impairment. Sleep. 2024;47:zsae051. 38394454 10.1093/sleep/zsae051 PMC11168761

[47] Eide PK , Vinje V , Pripp AH , Mardal K‐A , Ringstad G . Sleep deprivation impairs molecular clearance from the human brain. Brain. 2021;144:863‐874. 33829232 10.1093/brain/awaa443

[48] Hablitz LM , Vinitsky HS , Sun Q , et al. Increased glymphatic influx is correlated with high EEG delta power and low heart rate in mice under anesthesia. Sci Adv. 2019;5:eaav5447. 30820460 10.1126/sciadv.aav5447 PMC6392807

[49] Sharon O , Zhelezniakov V , Gat Y , et al. Slow wave synchrony during NREM sleep tracks cognitive impairment in prodromal Alzheimer's disease. Alzheimers Dement. 2025;21:e70247. 40399753 10.1002/alz.70247 PMC12094885

[50] Parrino L , Halasz P , Tassinari CA , Terzano MG . CAP, epilepsy and motor events during sleep: the unifying role of arousal. Sleep Med Rev. 2006;10:267‐285. 16809057 10.1016/j.smrv.2005.12.004

[51] Terzano MG , Parrino L , Rosa A , Palomba V , Smerieri A . CAP and arousals in the structural development of sleep: an integrative perspective. Sleep Med. 2002;3:221‐229. 14592211 10.1016/s1389-9457(02)00009-6

[52] Winer JR , Mander BA , Helfrich RF , et al. Sleep as a potential biomarker of tau and *β*‐amyloid burden in the human brain. J Neurosci. 2019;39:6315‐6324. 31209175 10.1523/JNEUROSCI.0503-19.2019 PMC6687908

[53] Tononi G , Cirelli C . Sleep and the price of plasticity: from synaptic and cellular homeostasis to memory consolidation and integration. Neuron. 2014;81:12‐34. 24411729 10.1016/j.neuron.2013.12.025 PMC3921176

[54] Zavecz Z , Shah VD , Murillo OG , et al. NREM sleep as a novel protective cognitive reserve factor in the face of Alzheimer's disease pathology. BMC Med. 2023;21:156. 37138290 10.1186/s12916-023-02811-z PMC10155344

[55] Mander BA , Winer JR , Walker MP . Sleep and human aging. Neuron. 2017;94:19‐36. 28384471 10.1016/j.neuron.2017.02.004 PMC5810920

[56] Himali JJ , Baril A‐A , Cavuoto MG , et al. Association between slow‐wave sleep loss and incident dementia. JAMA Neurol. 2023;80:1326‐1333. 37902739 10.1001/jamaneurol.2023.3889 PMC10616771

[57] Hauglund NL , Andersen M , Tokarska K , et al. Norepinephrine‐mediated slow vasomotion drives glymphatic clearance during sleep. Cell. 2025;188:606‐622. e17. 39788123 10.1016/j.cell.2024.11.027 PMC12340670

[58] André C , Champetier P , Rehel S , et al. Rapid eye movement sleep, neurodegeneration, and amyloid deposition in aging. Ann Neurol. 2023;93:979‐990. 36641644 10.1002/ana.26604

[59] Jin J , Chen J , Cavaillès C , et al. Association of rapid eye movement sleep latency with multimodal biomarkers of Alzheimer's disease. Alzheimers Dement. 2025;21:e14495. 39868572 10.1002/alz.14495 PMC11848184

[60] André C , Martineau‐Dussault M‐È , Baril A‐A , et al. Reduced rapid eye movement sleep in late middle‐aged and older apolipoprotein E ɛ 4 allele carriers. Sleep. 2024;47:zsae094. 38634644 10.1093/sleep/zsae094 PMC11236949

[61] André C , Martineau‐Dussault M‐È , Daneault V , et al. REM sleep is associated with the volume of the cholinergic basal forebrain in aMCI individuals. Alzheimers Res Ther. 2023;15:151. 37684650 10.1186/s13195-023-01265-y PMC10485959

[62] Lew CH , Petersen C , Neylan TC , Grinberg LT. Tau‐driven degeneration of sleep‐and wake‐regulating neurons in Alzheimer's disease. Sleep Med Rev. 2021;60:101541. 34500400 10.1016/j.smrv.2021.101541 PMC8862638

[63] Van Erum J , Van Dam D , De Deyn PP . Alzheimer's disease: neurotransmitters of the sleep‐wake cycle. Neurosci Biobehav Rev. 2019;105:72‐80. 31377219 10.1016/j.neubiorev.2019.07.019

[64] Mogavero MP , Godos J , Grosso G , Caraci F , Ferri R . Rethinking the role of orexin in the regulation of REM sleep and appetite. Nutrients. 2023;15:3679. 37686711 10.3390/nu15173679 PMC10489991

[65] Liguori C , Romigi A , Nuccetelli M , et al. Orexinergic system dysregulation, sleep impairment, and cognitive decline in Alzheimer disease. JAMA Neurol. 2014;71:1498‐1505. 25322206 10.1001/jamaneurol.2014.2510

[66] Leng Y , Musiek ES , Hu K , Cappuccio FP , Yaffe K . Association between circadian rhythms and neurodegenerative diseases. Lancet Neurol. 2019;18:307‐318. 30784558 10.1016/S1474-4422(18)30461-7 PMC6426656

[67] Wang C , Holtzman DM . Bidirectional relationship between sleep and Alzheimer's disease: role of amyloid, tau, and other factors. Neuropsychopharmacology. 2020;45:104‐120. 31408876 10.1038/s41386-019-0478-5 PMC6879647

[68] Kegyes‐Brassai AC , Pierson‐Bartel R , Bolla G , Kamondi A , Horvath AA . Disruption of sleep macro‐and microstructure in Alzheimer's disease: overlaps between neuropsychology, neurophysiology, and neuroimaging. Geroscience. 2024;47:1‐18. 39333449 10.1007/s11357-024-01357-z PMC12181553

