# Supplementary material for: Cyclic alternating pattern in sleep electroencephalography as a novel predictor of dementia: A prospective study
Source: Alzheimers Dement. 2026 Apr 1;22(4):e71331. doi: 10.1002/alz.71331 (PMC13045341; doi:10.1002/alz.71331)
Supplement: Supplementary file 1 — Supporting Information [file ALZ-22-e71331-s001.docx]

**Cyclic alternating pattern in sleep electroencephalography as novel predictors of dementia: a prospective study**

Ying Zheng et al

**SUPPLEMENTARY TABLE AND FIGURES LEGENDS**

Supplementary Table 1. Percentage of missing values of the covariates.

Supplementary Table 2. Association between CAP and dementia incidence stratified by age

Supplementary Table 3. Association between CAP and dementia incidence stratified by BMI

Supplementary Table 4. Association between CAP and dementia incidence stratified by the existence of T2D

Supplementary Table 5. Association between CAP and dementia incidence stratified by the existence of hypertension

Supplementary Table 6. Associations between CAP and dementia incidence using a competing risk model

Supplementary Table 7. Associations between CAP and dementia incidence with additional adjustment for NREM percentage and AHI.

Supplementary Table 8. Summary correlation table of CAP features with conventional sleep parameters.

Supplementary Table 9. AUC of the machine learning model for dementia in the test set.

Supplementary Figure 1. Flowchart of participant enrolment.

Supplementary Figure 2. The weights of variables importance by SHAP values.

| **Supplementary Table 1. Percentage of missing values of the covariates.** | |
| --- | --- |
| **Missing Covariates** | **N (%)** |
| Alcohol | 12 (0.47) |
| BMI | 1 (0.04) |
| PASE score | 3 (0.12) |

| **Supplementary Table 2.** Association between CAP and dementia incidence stratified by age | | | |
| --- | --- | --- | --- |
|  | **Age** | | |
|  | <75 years | ≥75 year | ***P* _interaction_** |
| CAP rate (%) cap time/NREM time |  |  | 0.909 |
| T1 vs. T3 | 1.63 (0.87, 3.06) | 1.25 (0.79, 1.99) |  |
| T2 vs. T3 | 1.25 (0.67, 2.33) | 0.98 (0.60, 1.60) |  |
| CAP index (cap cycles/h) |  |  | 0.307 |
| T1 vs. T3 | 1.40 (0.75, 2.61) | 1.35 (0.84, 2.16) |  |
| T2 vs. T3 | 1.16 (0.63, 2.15) | 1.17 (0.72, 1.89) |  |
| No. of CAP sequences |  |  | 0.609 |
| T1 vs. T3 | 1.06 (0.58, 1.96) | 1.03 (0.65, 1.64) |  |
| T2 vs. T3 | 0.77 (0.40, 1.46) | 0.88 (0.54, 1.43) |  |
| CAP sequence duration, min |  |  | 0.846 |
| T1 vs. T3 | 1.21 (0.67, 2.19) | 1.26 (0.78, 2.05) |  |
| T2 vs. T3 | 0.96 (0.52, 1.78) | 1.32 (0.81, 2.16) |  |
| A1 index |  |  | 0.521 |
| T1 vs. T3 | 1.01 (0.56, 1.81) | 1.01 (0.63, 1.63) |  |
| T2 vs. T3 | 0.71 (0.38, 1.33) | 0.98 (0.61, 1.57) |  |
| A2 index |  |  | 0.667 |
| T1 vs. T3 | 1.85 (0.99, 3.45) | 2.08 (1.30, 3.35) |  |
| T2 vs. T3 | 1.37 (0.73, 2.59) | 0.97 (0.56, 1.68) |  |
| A3 index |  |  | 0.959 |
| T1 vs. T3 | 1.31 (0.72, 2.38) | 1.25 (0.77, 2.05) |  |
| T2 vs. T3 | 1.10 (0.58, 2.08) | 1.40 (0.88, 2.24) |  |
| A2+A3 index |  |  | 0.926 |
| T1 vs. T3 | 1.87 (1.00, 3.50) | 1.94 (1.17, 3.23) |  |
| T2 vs. T3 | 1.41 (0.74, 2.69) | 1.73 (1.04, 2.89) |  |
| Model was adjusted for age, ethnicity, BMI, education, TST, WASO, history of diabetes and hypertension, smoking status, alcohol consumption, PASE score, the use of antidepressants, benzodiazepines, and other sleep medications. | | | |

| **Supplementary Table 3.** Association between CAP and dementia incidence stratified by BMI | | | |
| --- | --- | --- | --- |
|  | **BMI** | | |
|  | <30.0 | ≥30.0 | ***P* _interaction_** |
| CAP rate (%) cap time/NREM time |  |  | 0.148 |
| T1 vs. T3 | 1.33 (0.89, 2.00) | 1.60 (0.60, 4.24) |  |
| T2 vs. T3 | 1.03 (0.68, 1.56) | 1.47 (0.53, 4.02) |  |
| CAP index (cap cycles/h) |  |  | 0.270 |
| T1 vs. T3 | 1.25 (0.83, 1.90) | 1.91 (0.73, 4.99) |  |
| T2 vs. T3 | 1.09 (0.72, 1.64) | 1.71 (0.61, 4.82) |  |
| No. of CAP sequences |  |  | 0.694 |
| T1 vs. T3 | 1.01 (0.67, 1.53) | 1.18 (0.48, 2.90) |  |
| T2 vs. T3 | 0.85 (0.56, 1.30) | 0.88 (0.33, 2.36) |  |
| CAP sequence duration, min |  |  | 0.025 |
| T1 vs. T3 | 1.07 (0.71, 1.63) | 1.75 (0.72, 4.29) |  |
| T2 vs. T3 | 1.24 (0.82, 1.86) | 0.86 (0.30, 2.50) |  |
| A1 index |  |  | 0.440 |
| T1 vs. T3 | 1.04 (0.70, 1.56) | 0.91 (0.37, 2.26) |  |
| T2 vs. T3 | 0.87 (0.58, 1.32) | 0.94 (0.38, 2.32) |  |
| A2 index |  |  | 0.733 |
| T1 vs. T3 | 1.92 (1.27, 2.89) | 2.32 (0.90, 5.97) |  |
| T2 vs. T3 | 1.15 (0.73, 1.80) | 1.16 (0.40, 3.39) |  |
| A3 index |  |  | 0.302 |
| T1 vs. T3 | 1.13 (0.74, 1.73) | 1.79 (0.72, 4.43) |  |
| T2 vs. T3 | 1.31 (0.87, 1.96) | 1.11 (0.41, 3.02) |  |
| A2+A3 index |  |  | 0.969 |
| T1 vs. T3 | 1.71 (1.11, 2.63) | 2.67 (0.97, 7.33) |  |
| T2 vs. T3 | 1.58 (1.03, 2.43) | 1.82 (0.61, 5.42) |  |
| Model was adjusted for age, ethnicity, BMI, education, TST, WASO, history of diabetes and hypertension, smoking status, alcohol consumption, PASE score, the use of antidepressants, benzodiazepines, and other sleep medications. | | | |

| **Supplementary Table 4.** Association between CAP and dementia incidence stratified by the existence of T2D | | | |
| --- | --- | --- | --- |
|  | Existence of T2D | | |
|  | No | Yes | ***P* _interaction_** |
| CAP rate (%) cap time/NREM time |  |  | 0.666 |
| T1 vs. T3 | 1.41 (0.95, 2.08) | 1.17 (0.33, 4.13) |  |
| T2 vs. T3 | 1.00 (0.66, 1.51) | 1.99 (0.60, 6.66) |  |
| CAP index (cap cycles/h) |  |  | 0.235 |
| T1 vs. T3 | 1.47 (0.99, 2.19) | 0.71 (0.23, 2.16) |  |
| T2 vs. T3 | 1.17 (0.78, 1.75) | 1.11 (0.36, 3.41) |  |
| No. of CAP sequences |  |  | 0.191 |
| T1 vs. T3 | 1.14 (0.77, 1.69) | 0.51 (0.17, 1.58) |  |
| T2 vs. T3 | 0.86 (0.57, 1.30) | 0.62 (0.20, 1.92) |  |
| CAP sequence duration, min |  |  | 0.793 |
| T1 vs. T3 | 1.24 (0.84, 1.85) | 1.06 (0.35, 3.19) |  |
| T2 vs. T3 | 1.17 (0.78, 1.75) | 1.02 (0.32, 3.30) |  |
| A1 index |  |  | 0.372 |
| T1 vs. T3 | 1.06 (0.72, 1.55) | 0.64 (0.19, 2.18) |  |
| T2 vs. T3 | 0.86 (0.58, 1.28) | 1.09 (0.37, 3.15) |  |
| A2 index |  |  | 0.356 |
| T1 vs. T3 | 1.87 (1.27, 2.76) | 3.61 (0.77, 16.85) |  |
| T2 vs. T3 | 1.04 (0.67, 1.61) | 2.07 (0.41, 10.36) |  |
| A3 index |  |  | 0.783 |
| T1 vs. T3 | 1.30 (0.87,1.96) | 0.94 (0.33, 2.66) |  |
| T2 vs. T3 | 1.40 (0.94, 2.08) | 0.51 (0.16, 1.65) |  |
| A2+A3 index |  |  | 0.666 |
| T1 vs. T3 | 1.95 (1.29, 2.97) | 1.30 (0.41, 4.06) |  |
| T2 vs. T3 | 1.65 (1.08, 2.53) | 1.18 (0.36, 3.86) |  |
| Model was adjusted for age, ethnicity, BMI, education, TST, WASO, history of diabetes and hypertension, smoking status, alcohol consumption, PASE score, the use of antidepressants, benzodiazepines, and other sleep medications. | | | |

| **Supplementary Table 5.** Association between CAP and dementia incidence stratified by the existence of hypertension | | | |
| --- | --- | --- | --- |
|  | Existence of Hypertension | | |
|  | No | Yes | ***P* _interaction_** |
| CAP rate (%) cap time/NREM time |  |  | 0.033 |
| T1 vs. T3 | 1.03 (0.63, 1.66) | 2.13 (1.12, 4.03) |  |
| T2 vs. T3 | 0.95 (0.59, 1.54) | 1.52 (0.78, 2.97) |  |
| CAP index (cap cycles/h) |  |  | 0.097 |
| T1 vs. T3 | 1.02 (0.64, 1.65) | 2.03 (1.06, 3.89) |  |
| T2 vs. T3 | 0.79 (0.48, 1.29) | 2.03 (1.07, 3.87) |  |
| No. of CAP sequences |  |  | 0.028 |
| T1 vs. T3 | 0.82 (0.50, 1.35) | 1.29 (0.72, 2.33) |  |
| T2 vs. T3 | 0.88 (0.54, 1.43) | 0.84 (0.43, 1.61) |  |
| CAP sequence duration, min |  |  | 0.026 |
| T1 vs. T3 | 0.93 (0.57, 1.52) | 1.80 (0.98, 3.30) |  |
| T2 vs. T3 | 1.12 (0.70, 1.80) | 1.34 (0.70, 2.57) |  |
| A1 index |  |  | 0.662 |
| T1 vs. T3 | 0.98 (0.61, 1.56) | 1.05 (0.58, 1.92) |  |
| T2 vs. T3 | 0.75 (0.46, 1.23) | 1.15 (0.65, 2.06) |  |
| A2 index |  |  | 0.001 |
| T1 vs. T3 | 1.22 (0.76, 198) | 4.13 (2.07, 8.23) |  |
| T2 vs. T3 | 1.04 (0.64, 1.70) | 1.56 (0.71, 3.46) |  |
| A3 index |  |  | 0.012 |
| T1 vs. T3 | 0.84 (0.50, 1.39) | 2.07 (1.12, 3.85) |  |
| T2 vs. T3 | 1.23 (0.78, 1.95) | 1.41 (0.74, 2.70) |  |
| A2+A3 index |  |  | 0.073 |
| T1 vs. T3 | 1.45 (0.87, 2.40) | 2.57 (1.35, 4.90) |  |
| T2 vs. T3 | 1.66 (1.00, 2.73) | 1.71 (0.87, 3.35) |  |
| Model was adjusted for age, ethnicity, BMI, education, TST, WASO, history of diabetes and hypertension, smoking status, alcohol consumption, PASE score, the use of antidepressants, benzodiazepines, and other sleep medications. | | | |

| **Supplementary Table 6.** Associations between CAP and dementia incidence using a competing risk model | | | | |
| --- | --- | --- | --- | --- |
|  | No. of cases | No. of participants | SHR (95%CI) ^a^ |  |
| CAP rate (%) cap time/NREM time |  |  |  |  |
| T1 | 67 | 853 | 1.35 (0.93, 1.95) |  |
| T2 | 55 | 852 | 1.07 (0.73, 1.56) |  |
| T3 | 51 | 852 | 1 |  |
| P value for trend |  |  | 0.073 |  |
| CAP index (cap cycles/h) |  |  |  |  |
| T1 | 64 | 853 | 1.33 (0.91, 1.94) |  |
| T2 | 58 | 852 | 1.14 (0.78, 1.65) |  |
| T3 | 51 | 852 | 1 |  |
| P value for trend |  |  | 0.212 |  |
| No. of CAP sequences |  |  |  |  |
| T1 | 65 | 915 | 1.02 (0.70, 1.48) |  |
| T2 | 47 | 792 | 0.83 (0.56, 1.22) |  |
| T3 | 61 | 850 | 1 |  |
| P value for trend |  |  | 0.375 |  |
| CAP sequence duration, min |  |  |  |  |
| T1 | 65 | 854 | 1.21 (0.83, 1.76) |  |
| T2 | 56 | 851 | 1.14 (0.78, 1.66) |  |
| T3 | 52 | 852 | 1 |  |
| P value for trend |  |  | 0.032 |  |
| A1 index |  |  |  |  |
| T1 | 59 | 853 | 0.99 (0.68, 1.43) |  |
| T2 | 54 | 852 | 0.88 (0.61, 1.27) |  |
| T3 | 60 | 852 | 1 |  |
| P value for trend |  |  | 0.600 |  |
| A2 index |  |  |  |  |
| T1 | 82 | 853 | 1.98 (1.37, 2.87) |  |
| T2 | 48 | 852 | 1.12 (0.74, 1.70) |  |
| T3 | 43 | 852 | 1 |  |
| P value for trend |  |  | <0.001 |  |
| A3 index |  |  |  |  |
| T1 | 60 | 853 | 1.25 (0.86, 1.82) |  |
| T2 | 63 | 852 | 1.25 (0.86, 1.83) |  |
| T3 | 50 | 852 | 1 |  |
| P value for trend |  |  | 0.123 |  |
| A2 + A3 index |  |  |  |  |
| T1 | 70 | 853 | 1.84 (1.25, 2.73) |  |
| T2 | 63 | 852 | 1.58 (1.06, 2.36) |  |
| T3 | 40 | 852 | 1 |  |
| P value for trend |  |  | 0.007 |  |
| ^a^ Model was adjusted for age, ethnicity, BMI, education, TST, WASO, history of diabetes and hypertension, smoking status, alcohol consumption, PASE score, the use of antidepressants, benzodiazepines, and other sleep medications. | | | |  |

| **Supplementary Table 7.** Associations between CAP and dementia incidence with additional adjustment for NREM percentage and AHI. | | | | |
| --- | --- | --- | --- | --- |
|  | No. of cases | No. of participants | HR (95%CI) |  |
| CAP rate (%) cap time/NREM time |  |  |  |  |
| T1 | 67 | 853 | 1.34 (0.92, 1.95) |  |
| T2 | 55 | 852 | 1.07 (0.73, 1.58) |  |
| T3 | 51 | 852 | 1 |  |
| P value for trend |  |  | 0.077 |  |
| CAP index (cap cycles/h) |  |  |  |  |
| T1 | 64 | 853 | 1.32 (0.90, 1.92) |  |
| T2 | 58 | 852 | 1.15 (0.79, 1.68) |  |
| T3 | 51 | 852 | 1 |  |
| P value for trend |  |  | 0.209 |  |
| No. of CAP sequences |  |  |  |  |
| T1 | 65 | 915 | 1.08 (0.74, 1.58) |  |
| T2 | 47 | 792 | 0.88 (0.60, 1.30) |  |
| T3 | 61 | 850 | 1 |  |
| P value for trend |  |  | 0.206 |  |
| CAP sequence duration, min |  |  |  |  |
| T1 | 65 | 854 | 1.21 (0.83, 1.75) |  |
| T2 | 56 | 851 | 1.16 (0.79, 1.69) |  |
| T3 | 52 | 852 | 1 |  |
| P value for trend |  |  | 0.028 |  |
| A1 index |  |  |  |  |
| T1 | 59 | 853 | 1.00 (0.69, 1.44) |  |
| T2 | 54 | 852 | 0.89 (0.61, 1.29) |  |
| T3 | 60 | 852 | 1 |  |
| P value for trend |  |  | 0.603 |  |
| A2 index |  |  |  |  |
| T1 | 82 | 853 | 2.07 (1.31, 3.04) |  |
| T2 | 48 | 852 | 1.17 (0.77, 1.77) |  |
| T3 | 43 | 852 | 1 |  |
| P value for trend |  |  | < 0.001 |  |
| A3 index |  |  |  |  |
| T1 | 60 | 853 | 1.23 (0.84, 1.81) |  |
| T2 | 63 | 852 | 1.26 (0.87, 1.84) |  |
| T3 | 50 | 852 | 1 |  |
| P value for trend |  |  | 0.132 |  |
| A2 + A3 index |  |  |  |  |
| T1 | 70 | 853 | 1.86 (1.25, 2.76) |  |
| T2 | 63 | 852 | 1.62 (1.09, 2.41) |  |
| T3 | 40 | 852 | 1 |  |
| P value for trend |  |  | 0.006 |  |
| ^a^ Model was adjusted for age, ethnicity, BMI, education, TST, WASO, history of diabetes and hypertension, smoking status, alcohol consumption, PASE score, the use of antidepressants, benzodiazepines, and other sleep medications. | | | |  |

**Supplementary Table 8.** Summary correlation table of CAP features with conventional sleep parameters.

|  | CAP rate | CAP index | Number of CAP sequences | CAP sequence duration | A1 index | A2 index | A3 index | A2 + A3 index |
| --- | --- | --- | --- | --- | --- | --- | --- | --- |
| TST | r=-0.068 | r=-0.120 | r=0.290 | r=0.003 | r=-0.088 | r=-0.002 | r=-0.056 | r=-0.052 |
| WASO | r=-0.032 | r=0.002 | r=-0.085 | r=-0.080 | r=-0.004 | r=-0.041 | r=-0.026 | r=-0.034 |
| AHI | r=0.068 | r=0.067 | r=0.076 | r=0.048 | r=-0.133 | r=0.147 | r=0.148 | r=0.177 |
| NREM percentage | r=-0.009 | r=-0.002 | r=0.12 | r=-0.019 | r=-0.050 | r=0.058 | r=-0.002 | r=0.017 |

| **Supplementary Table 9.** AUC of the machine learning model for dementia in the test set. | |
| --- | --- |
|  | AUC |
| CAP rate | 0.538 |
| CAP index | 0.530 |
| No. of CAP sequences | 0.550 |
| CAP sequence duration | 0.514 |
| A1 index | 0.564 |
| A2 index | 0.622 |
| A3 index | 0.546 |
| A2+A3 index | 0.565 |
| AUC, ROC curve area under the curve |  |

**Supplementary Figure 1. Flowchart of participant enrolment.**


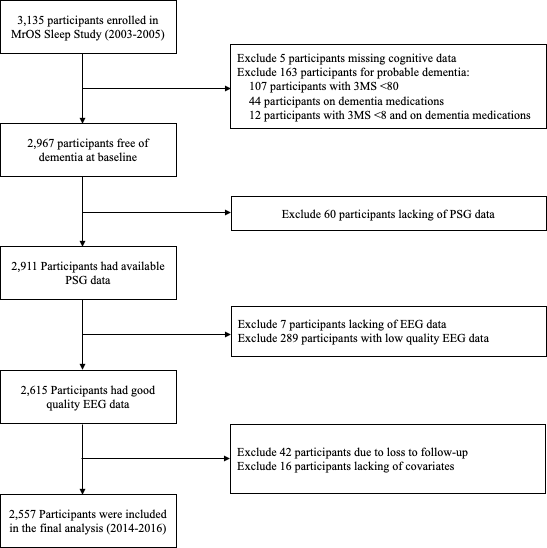


**Supplementary Figure 2. The weights of variables importance by SHAP values.**

| **a** | **b** |
| --- | --- |
| 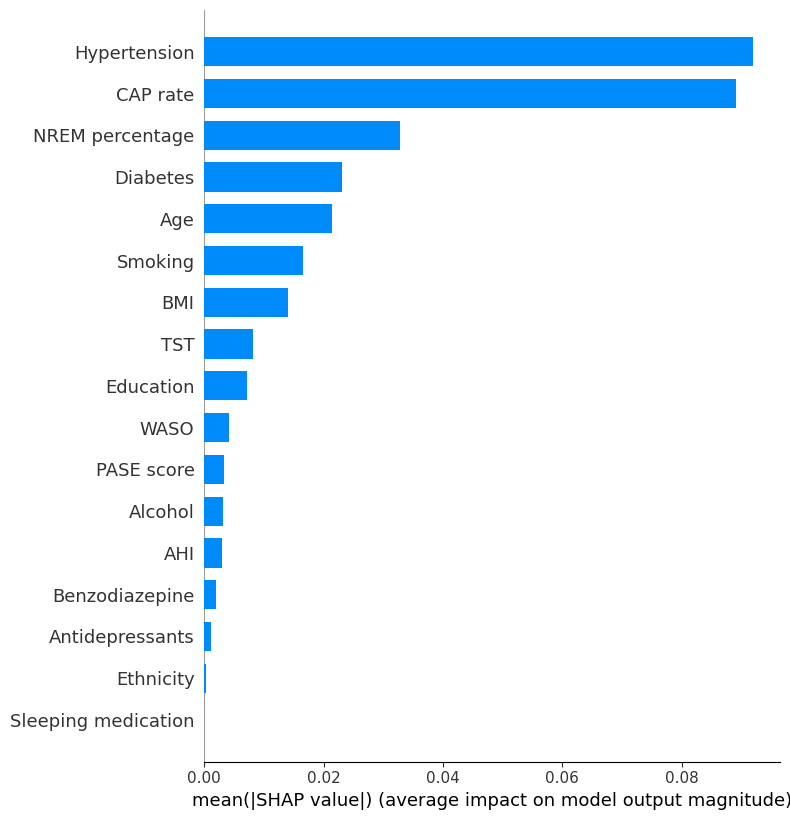 | 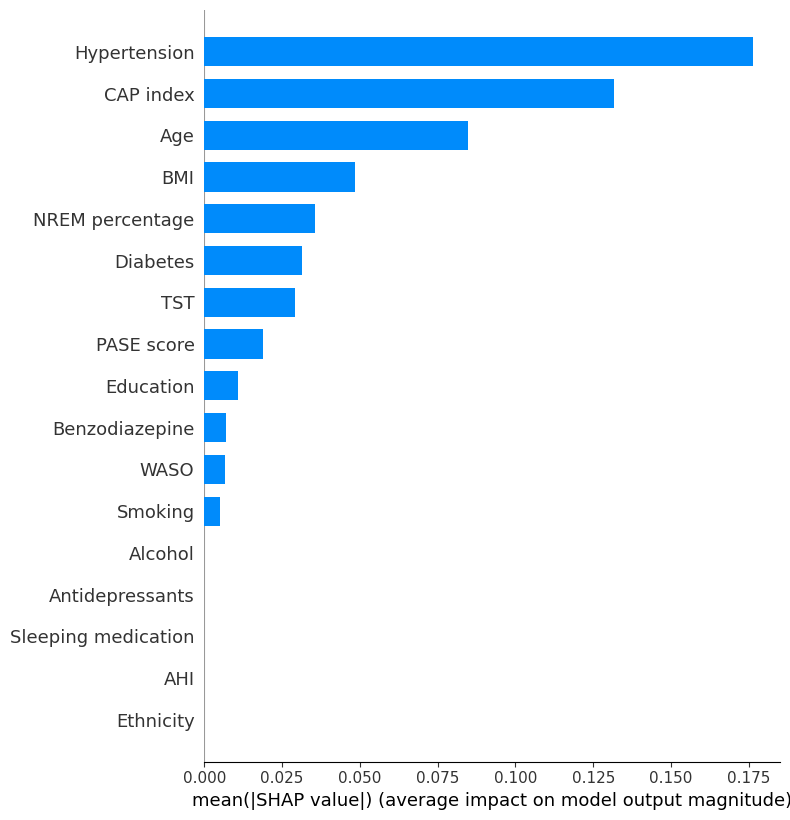 |
| **c** **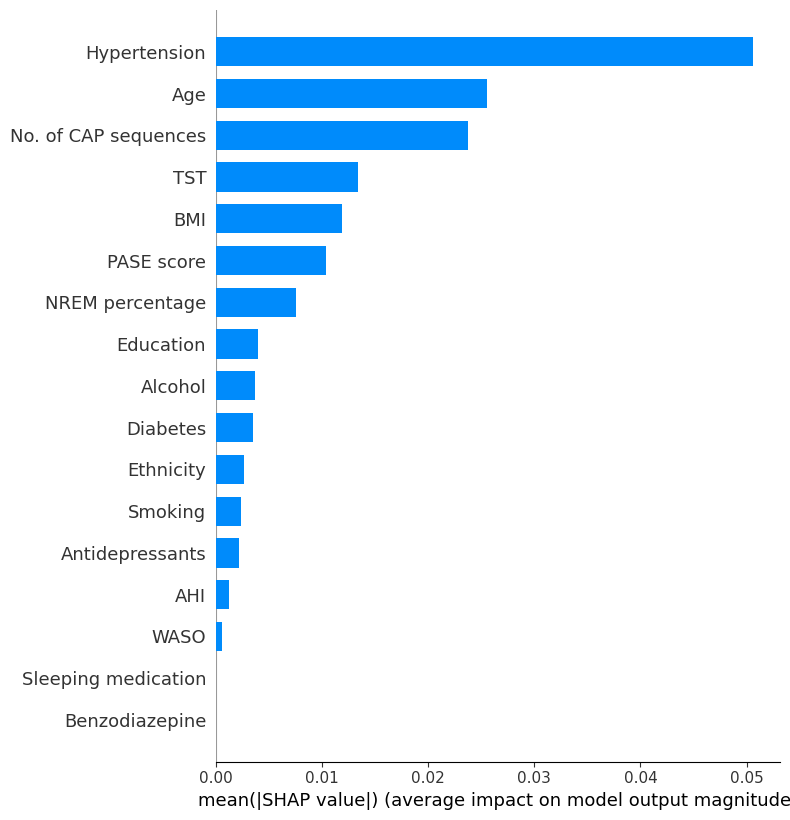** | **d** **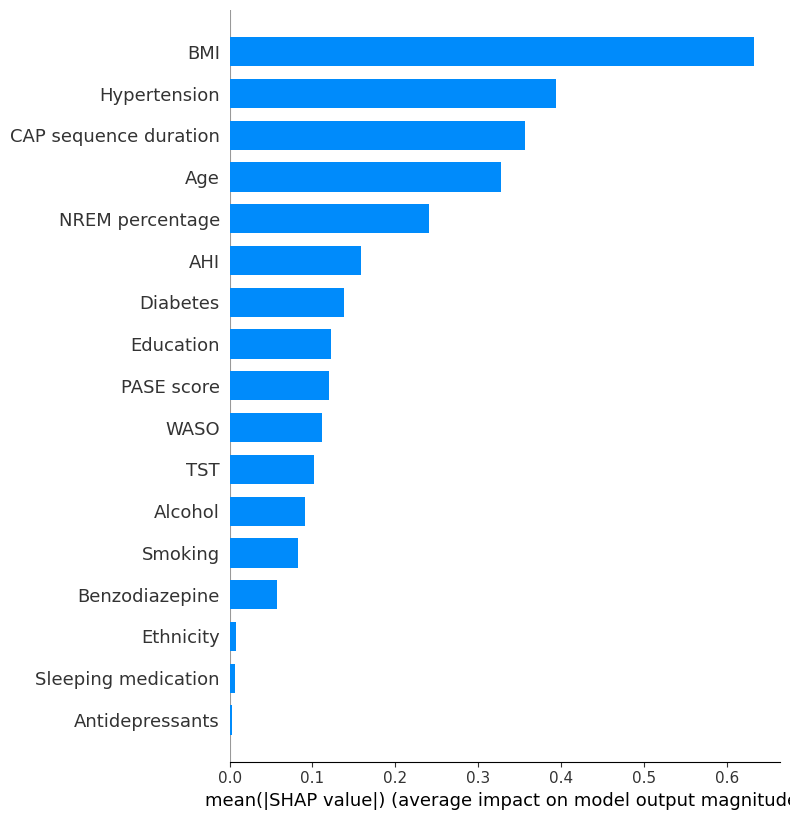** |
|  |  |
| **e** | **f** |
| 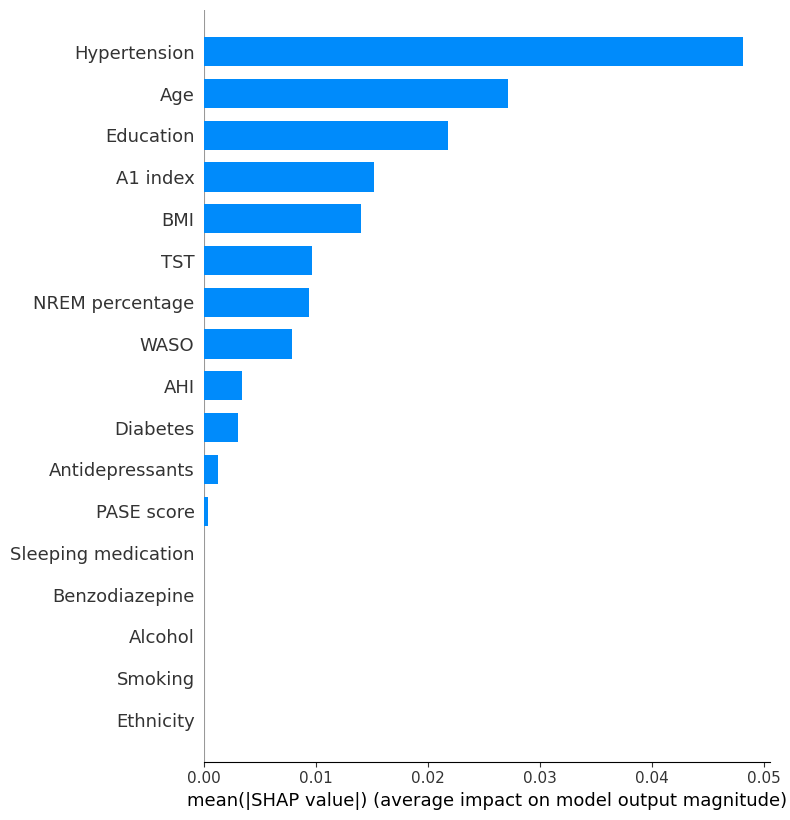 | 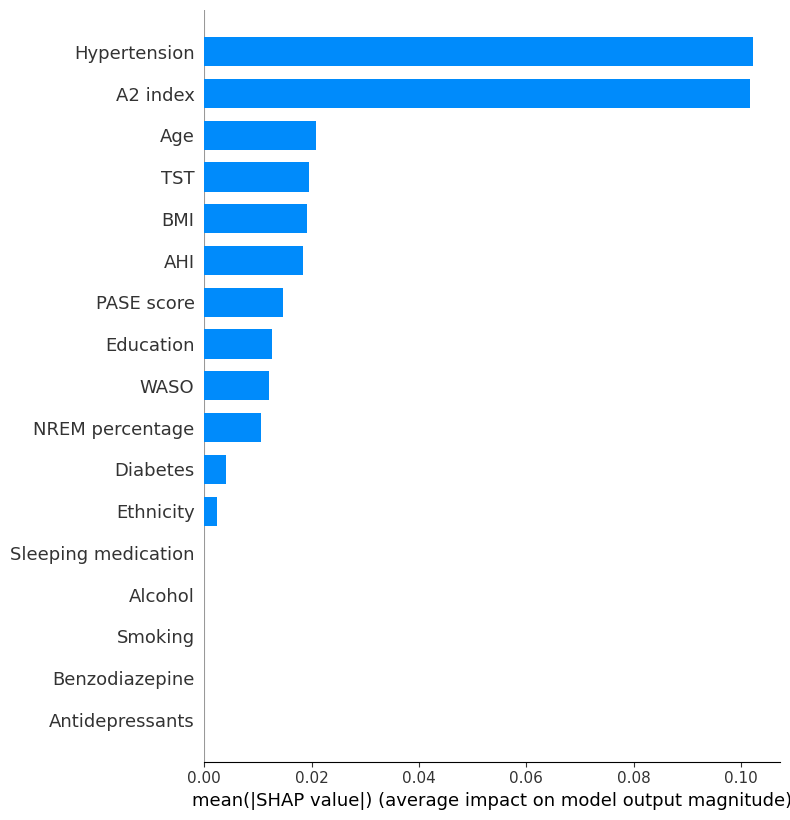 |
| **g** | **h** |
| 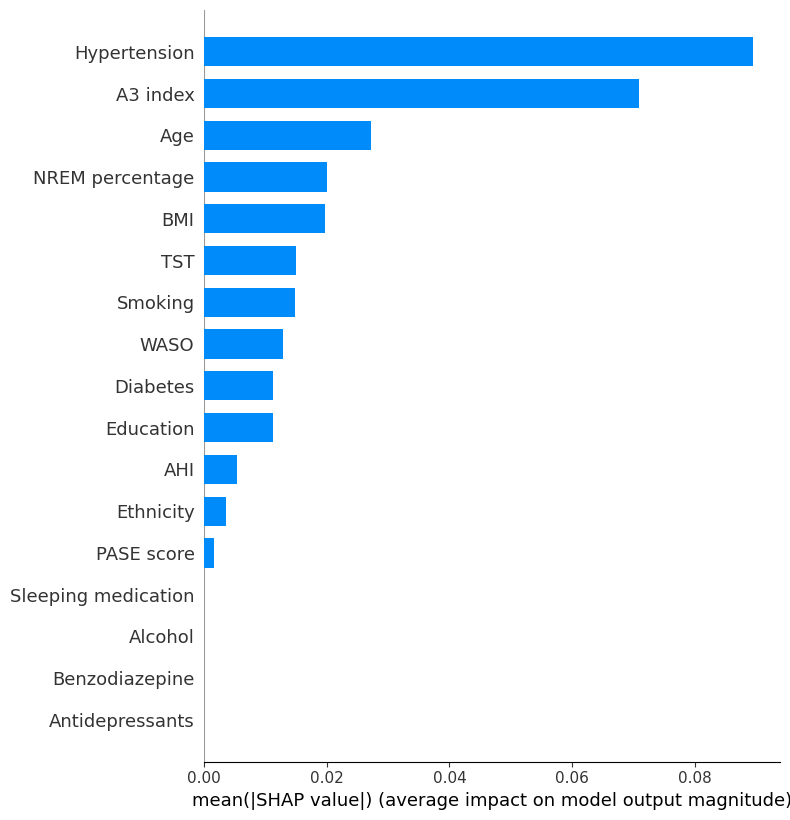 | 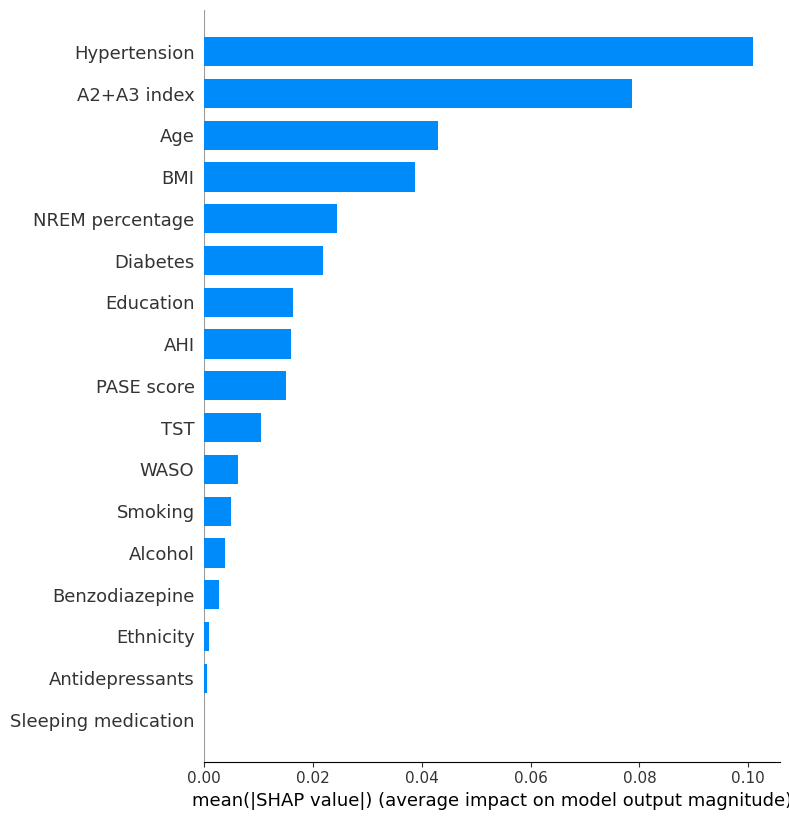 |

**a** CAP rate; **b** CAP index; **c** No. of CAP sequences; **d** CAP sequence duration; **e** A1 index; **f** A2 index; **g** A3 index; **h** A2+A3 index. Features are ordered based on their cumulative effect on model output. BMI = body mass index; PASE = Physical Activity Scale for the Elderly; TST = total sleep duration; WASO = wake after sleep onset.
